# Customized Toric Intraocular Lens Implantation in Eyes with Cataract and Corneal Astigmatism after Deep Anterior Lamellar Keratoplasty: A Prospective Study

**DOI:** 10.1155/2018/1649576

**Published:** 2018-07-03

**Authors:** Domenico Schiano Lomoriello, Giacomo Savini, Kristian Naeser, Rossella Maria Colabelli-Gisoldi, Valeria Bono, Augusto Pocobelli

**Affiliations:** ^1^IRCCS Fondazione G.B. Bietti, Rome, Italy; ^2^Regions Hospital Randers, Randers, Denmark; ^3^Azienda Ospedaliera San Giovanni-Addolorata, Rome, Italy

## Abstract

**Purpose:**

To investigate the effectiveness of toric intraocular lenses (IOLs) for treating corneal astigmatism in patients with cataract and previous deep anterior lamellar keratoplasty (DALK).

**Setting:**

San Giovanni-Addolorata Hospital, Rome, Italy.

**Design:**

Prospective interventional case series.

**Methods:**

Patients undergoing cataract surgery after DALK for keratoconus were enrolled. Total corneal astigmatism (TCA) was assessed by a rotating Scheimpflug camera combined with Placido-disk corneal topography (Sirius; CSO, Firenze, Italy). A customized toric IOL (FIL 611 T, Soleko, Rome, Italy) was implanted in all eyes. One year postoperatively, refraction was measured, the IOL position was recorded, and vectorial and nonvectorial analyses were performed to evaluate the correction of astigmatism.

**Results:**

Ten eyes of 10 patients were analyzed. The mean preoperative TCA magnitude was 4.92 ± 1.99 diopters (D), and the mean cylinder of the IOL was 6.18 ± 2.44. After surgery, the difference between the planned axis of orientation of the IOL and the observed axis was ≤10° in all eyes. The mean surgically induced corneal astigmatism was 0.35 D at 20°. The mean postoperative refractive astigmatism power was 1.13 ± 0.94 D; with respect to preoperative TCA, the reduction was statistically significant (*p* < 0.0001). The mean change in astigmatism power was 3.80 ± 1.60 D, corresponding to a correction of 77% of preoperative TCA power. Nine eyes out of 10 had a postoperative refractive astigmatism power ≤ 2D.

**Conclusions:**

Toric IOLs can effectively correct corneal astigmatism in eyes with previous DALK. The predictability of cylinder correction is partially lowered by the variability of the surgically induced changes of TCA. This trial is registered with NCT03398109.

## 1. Introduction

Tissue transparency is the main factor affecting a successful outcome of corneal grafts, but a good postoperative refraction is also essential to achieve patients' satisfaction [1]. High astigmatism is the most common cause of unsatisfactory vision after keratoplasty when the transplanted cornea is transparent [1, 2]. Spectacles and contact lenses can be adopted for regular low-grade astigmatism but lead to poor vision or are not tolerated in cases with high astigmatism secondary to corneal transplantation [3]. Among surgical procedures, arcuate keratotomy reduces postkeratoplasty astigmatism, but the results of this technique are often unpredictable [3, 4]. Both photorefractive keratectomy (PRK) and laser in situ keratomileusis (LASIK) are effective, but not suitable for all patients who underwent corneal transplant, due to the risk of complications and the unreliable refractive outcomes [3, 5, 6]. Intrastromal corneal ring segments implantation could be also a viable option for effective correction of post-DALK astigmatism [7]. However, patients previously operated by penetrating keratoplasty (PK) presenting cataract can benefit of a phacoemulsification with toric intraocular lens (IOL) implants with a reduction of the postkeratoplasty astigmatism [8–15]. In the last decade, several studies showed that deep anterior lamellar keratoplasty (DALK) should be preferred to PK in patients with corneal pathology and healthy endothelium [16–21] because DALK leads to comparable visual outcomes and lower rates of intraoperative and postoperative complications. However, an extensive research on the major biomedical databases (PubMed, Scopus, ScienceDirect, Google Scholar) failed to identify studies investigating the implantation of toric IOLs after DALK, with the only exception of a case report in a patient with subluxated cataract after DALK [22]. The preoperative corneal cylinder in these eyes is often so high that standard manufactured toric IOL powers are insufficient. Therefore, the purpose of this study was to assess the efficacy of custom-made toric intraocular lens implantation in patients with simultaneous post-DALK high corneal astigmatism and cataract.

## 2. Methods

This study was designed as a prospective, noncomparative interventional case series. It has been approved by the institute review board and the regional ethical committee to adhere to the principles of the Declaration of Helsinki. All enrolled patients attended the corneal service of San Giovanni-Addolorata Hospital, where they had undergone DALK for keratoconus between January 2007 and December 2014. They reported a recent visual decrease in the transplanted eye due to cataract, significantly affecting the visual acuity. Corneal suture removal had been performed in all cases at least 1 year before cataract surgery. Corneal astigmatism was stable at least since 6 months before the cataract surgery in all patients.

### 2.1. Preoperative Examinations and Toric IOL Power Calculation

All patients underwent a comprehensive preoperative assessment that included uncorrected distance visual acuity (UDVA), distance-corrected visual acuity (DCVA), slit-lamp examination, optical biometry by means of partial coherence interferometry (Carl Zeiss IOLMaster V.5.4.1, Carl Zeiss Meditec, Jena, Germany), corneal tomography by means of a rotating Scheimpflug camera combined with a Placido-disc corneal topographer (Sirius; CSO, Firenze, Italy) and specular microscope (Perseus; CSO, Firenze, Italy). The magnitude and direction of the IOL cylinder were based on total corneal astigmatism (TCA) measurement, as calculated by the Sirius with ray-tracing over a 3 mm diameter. The instrument software uses all the traced rays to calculate the wavefront error, that is, the difference between the measured wavefront and an ideal spherical wavefront. The wavefront error, including astigmatism magnitude and axis, is then fitted using Zernike polynomials, as previously described [23]. TCA values were provided to the IOL manufacturer (Soleko S.p.A., Rome, Italy) that calculated the required IOL cylinder by means of proprietary software. The toric IOL was customized and manufactured in 0.25 D cylinder steps. The implanted toric IOL was a FIL 611 T (Soleko S.p.A., Rome, Italy), whose material is afoldable acrylate with 25% water content. The IOL has a plate-haptic design, a 6 mm optic diameter and 11.80 mm overall length. The cylindrical correction is directly built on the posterior IOL surface (“real axis technology”), so that, once implanted in the capsular bag, the reference marks on the toric IOL have to be aligned to the 0–180° axis.

### 2.2. Surgical Procedure

Both DALK and cataract surgery were carried out by the same experienced surgeon (A.P.). DALK was performed with “big-bubble” technique with achievement of a big bubble in all cases [16, 17, 24, 25]. All patients received a routine phacoemulsification surgery under topical anesthesia. Limbal marks were made at 180 degree before the surgery with the patients in a sitting position focusing at distance. Phacoemulsification was performed with the Infiniti OZil (Alcon, Forth Worth, TX) through a 2.75 mm temporal clear cornea incision (CCI). All CCIs were performed as limbal as possible, avoiding the corneal graft-host junction. No sutures were applied at the end of the surgeries.

### 2.3. Postoperative Evaluations

At 12 months postoperatively, patients underwent UDVA and DCVA measurements, slit-lamp examination under mydriasis (in order to record the orientation of the IOL), corneal tomography, and specular microscopy. The difference between the preoperative and postoperative TCA was used to calculate the surgically induced corneal astigmatism (SICA).

### 2.4. Vector Analysis of Astigmatism

Vector analysis according to Naeser [26] was used to calculate the surgically induced corneal astigmatism (SICA), the difference in TCA between preoperative and postoperative measurements, and the error in refractive astigmatism (ERA), defined as the difference between the observed and the targeted postoperative refractive astigmatism. Briefly, the net astigmatism (*M* at *α*), where *M* is the astigmatic magnitude in diopters (D) and *α* is the astigmatic direction in degrees, was transformed into two polar values in units of diopters:

  Meridional power = polar value along the reference meridian

(1) $$\begin{array}{c} \Phi \;\text{degrees}=\text{KP⁡}\left(\Phi \right)=M\text{⁡}\cos\left(\left.2\text{⁡}*\left(\alpha -\Phi \right)\right)\right.. \end{array}$$

  Torsional power = polar value along the meridian

(2) $$\begin{array}{c} \left(\Phi +45\right)\;\text{degrees}=\text{KP⁡}\left(\Phi +45\right)=M\text{⁡}\sin\left(2\text{⁡}*\left(\alpha -\Phi \right)\right). \end{array}$$

For calculation of SICA, the reference plane is the surgical meridian in zero degrees, reducing (1) and (2) to

  Meridional power = polar value along zero degrees

(3) $$\begin{array}{c} \text{KP }\left(0\right)=M\text{⁡}\cos\left(2\text{⁡}*\text{⁡}\alpha \right). \end{array}$$

  Torsional power = polar value along the meridian 45°

(4) $$\begin{array}{c} \text{KP⁡}\left(45\right)=M\text{⁡}\sin\left(2\text{⁡}*\text{⁡}\alpha \right). \end{array}$$

KP(0) is negative for a flattening and positive for a steepening of the surgical meridian along zero degrees.

KP(45) is negative for a clockwise and positive for a counterclockwise rotation of the cylinder median in relation to the horizontal meridian. Refractive data were transformed from the vertex to the corneal plane and then further to polar values. Meridional and torsional powers were reconverted to the usual net cylinder notation by means of the following general equations [26]:

(5) $$\begin{array}{c} M=\sqrt{\text{KP}{\left(\Phi \right)}^{2}+\text{KP}{\left(\Phi +45\right)}^{2}},\\ \alpha =\mathrm{arc}\;\tan\left(\frac{M-\text{KP}\left(\Phi \right)}{\text{KP}\left(\Phi +45\right)}\right)+\Phi . \end{array}$$

Equation (5) was used also to calculate the ERA, according to two models, as previously reported [27]:

- Model 1 is based on preoperative corneal measurements, mean observed SICA, observed IOL axis position, and IOL toric power at the corneal plane (as calculated by the manufacturer). In this model, the reference meridian Φ is the target TCA, defined as the vector sum of the preoperative TCA and the SICA.
- Model 2 is based on postoperative corneal measurements, observed IOL axis position and IOL toric power at the corneal plane (as calculated by the manufacturer). In this model, the reference meridian Φ is the postoperative TCA.

The interpretation of ERA was identical for both models. KP(Φ) is negative for an overcorrection and positive for an undercorrection along the reference meridian. KP(Φ + 45) is negative for a clockwise and positive for a counterclockwise rotation of the cylinder median in relation to the reference meridian.

### 2.5. Statistical Analysis

All statistical tests were performed using Instat (version 3.10 for Windows, GraphPad Software, La Jolla, CA). The Wilcoxon matched pairs signed-ranks test was used to compare the mean values. The Wilcoxon rank sum test was used to investigate the difference of one sample with respect to zero. A *p* value < 0.05 was considered statistically significant.

## 3. Results

Ten eyes of 10 patients (6 men) were enrolled. Mean age was 67.1 ± 7.3 years (range: 56 to 76 years). Cataract surgery and toric IOL implantation were carried out in all cases with no complications. The mean IOL power was 10.9 ± 7.9D (range: −1.25 to +21.5), and the mean cylinder at the IOL plane was 6.18 ± 2.44D (range: +3.25 to +11.5). The mean follow-up after cataract surgery was 15.9 ± 8.9 months (range: 7 to 30 months). Postoperatively, at the end of follow-up, the 0–180° reference marks on the IOL were oriented on average at 175.6° (range: 170–5°), and in 100% of eyes, the difference between the planned axis of orientation and the observed axis was ≤10°. The preoperative and postoperative parameters and the spherical equivalent power and cylinder power of the implanted IOL are reported in Table 1. Postoperatively, both UDVA and DCVA improved significantly with respect to the corresponding preoperative values. UDVA improved from 1.53 ± 0.54 to 0.29 ± 0.13 LogMAR (*p*=0.0039), and DCVA improved from 0.55 ± 0.29 to 0.14 ± 0.12 LogMAR (*p*=0.002). The postoperative refraction spherical equivalent was −0.16 ± 0.84 D.

### 3.1. Surgically Induced Corneal Astigmatism (SICA)

Surgery produced an average 0.27 ± 1.16 D steepening along the incision meridian and an average 0.23 ± 1.44 D counterclockwise rotation over the horizontal surgical meridian (Table 2). These values correspond to a mean SICA of 0.35 D at 20°. No statistically significant differences were found between the average preoperative and postoperative values of meridional and torsional power. Surgery had little effect on the orientation of the steepest TCA axis, since a change in axis orientation of the steepest meridian >10° was observed just in 1 eye.

### 3.2. Error in Refractive Astigmatism

**Figure 1:**
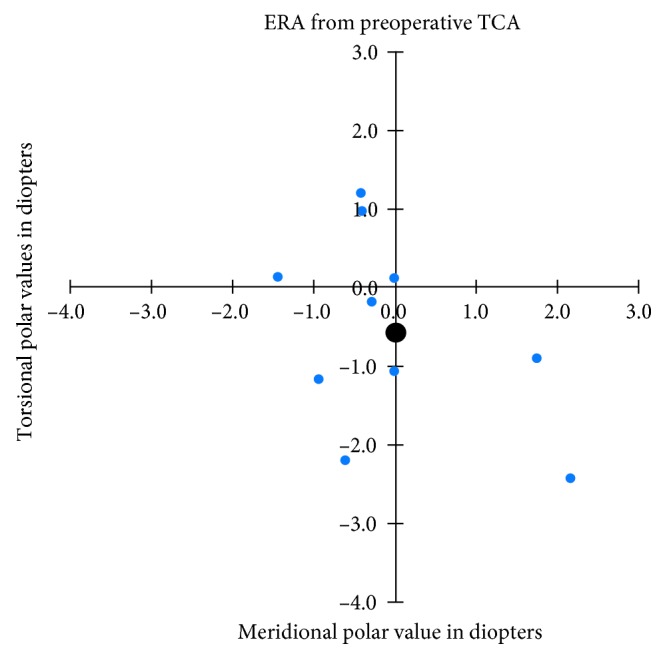
The error in refractive astigmatism (ERA) in calculations based on preoperatively measured total corneal astigmatism (TCA). *X*-axis: ERA expressed as the meridional polar value. *Y*-axis: ERA expressed as the torsional polar value. The small colored dots indicate the individual observations. The large black dots indicate the combined mean (centroid).

**Figure 2:**
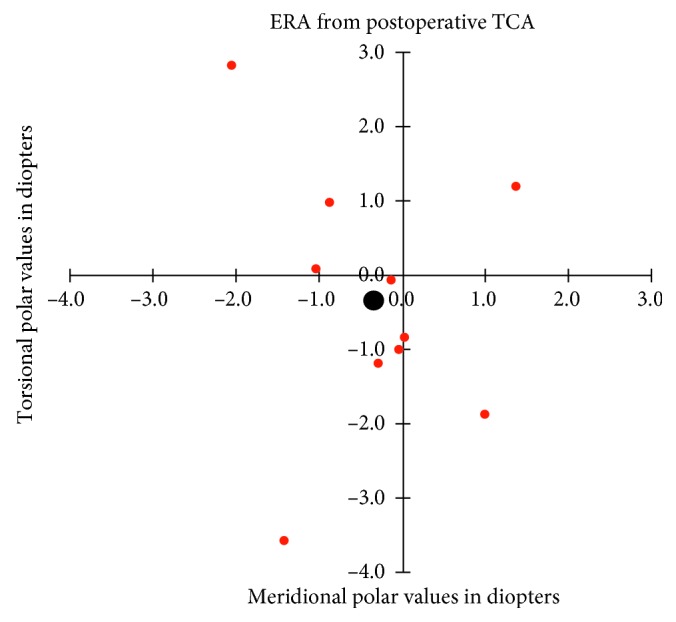
The error in refractive astigmatism (ERA) in calculations based on postoperatively measured total corneal astigmatism (TCA). *X*-axis: ERA expressed as the meridional polar value. *Y*-axis: ERA expressed as the torsional polar value. The small colored dots indicate the individual observations. The large black dots indicate the combined mean (centroid).

The absolute mean postoperative refractive astigmatism power (at the corneal plane) was 1.13 ± 0.94 D (range: 0–3 D). Compared to the preoperative TCA power 4.92 ± 1.99 D, the resulting average 3.80 ± 1.60 D reduction was statistically significant (*p* < 0.002) and allowed us to correct 77 ± 16% of the preoperative TCA magnitude (range 48.9 to 100 %). Nine eyes out of 10 had a postoperative refractive astigmatism power ≤2 D, whereas preoperatively, TCA was ≥2 D in all eyes. Calculations of ERA meridional powers revealed small average overcorrections of 0.03 (±1.13) D and 0.36 (±1.04) D for measurements based on preoperative and postoperative TCA, respectively. Torsional powers disclosed average counterclockwise rotations amounting to 0.55 ± 1.22 D and 0.34 ± 1.79 D. None of these meridional and torsional average powers differed significantly from zero. Figures 1 and 2 show the distribution of individual and mean ERA based on both preoperative and postoperative TCA measurements. ERA mean absolute error (MAE) amounted to 1.43 ± 0.91 D and 1.69 ± 1.18 D for preoperative and postoperative TCA measurements, with no statistically significant difference between them. The postoperative endothelial cell reduction was 8% compared to preoperative values. No patients had postoperative corneal decompensation.

## 4. Discussion

Our results show that cataract extraction with toric IOL implantation is effective to reduce astigmatism and improve visual acuity in patients with previous DALK. Postoperative improvements of UDVA, CDVA, and astigmatism were all statistically significant. Previous studies reported the effectiveness of toric IOL s implantation in eyes with previous PK [8–15]. A retrospective study, recently published on a peer-reviewed but not indexed journal, describes good outcomes for toric IOLs in eyes with previous DALK [28]. Our data confirm these results, as shown by the significant improvement of UDVA and CDVA and add some interesting findings related to vector analysis (which was not carried out by Scorcia et al.) [28]. In our sample, the refractive predictability was good, as in 90% of patients, the postoperative refractive astigmatism was within 2 D and, on average, more than 75% of astigmatism magnitude was corrected. The good outcomes can be related to two factors at least. First, the toric IOL was customized, that is, manufactured in steps of 0.25 D, according to the axial length and corneal astigmatism values that we supplied preoperatively. Moreover, the cylinder power of the IOL could be manufactured to correct as many as more than 9 D, which is not possible with standard toric IOLs. Second, calculations were based on TCA rather than on KA, as the former has been shown to provide more accurate results [29]. Vector analysis showed that using both preoperative and postoperative TCA leads to a good predictability in the refractive outcome, as the average ERA was close to zero in both cases. The ERA standard deviations for meridional (±1.13D) and torsional (±D) powers should be compared to the approximate ± 0.60 D values for normal eyes [29]. The relatively large variability may depend on a lower repeatability of TCA measurements in eyes with irregular astigmatism. On the other hand, the predictability of astigmatism correction in these eyes can be reduced due to any irregular component of corneal astigmatism, which cannot be corrected by toric IOLs and by the variable SICA induced by the incision. In this regard, although vector analysis revealed a minimal average SICA (just 0.35 D at 20°), we should consider that this value is misleading, as opposite astigmatism is cancelled out by vector analysis. A more realistic value is provided by the range of the meridional (from −0.86 to +3.00 D) and torsional (from −2.00 to +2.42 D) power changes in corneal astigmatism, which highlight the risk of TCA changes induced by the incision. The SICA standard deviations for meridional and torsional power in normal eyes were recently reported as ± 0.40 D and ± 0.48 D [29], which is far less than the similar values of ± 1.16 D and ± 1.44 D in the present series. Usually, surgery induces a corneal flattening along the surgical meridian and a compensatory steepening along its orthogonal meridian, the so-called coupling effect [26]. In the present study, a 0.27 D steepening was observed. This average result was influenced by a 3.0 D corneal steepening in a single eye with a preoperative TCA of 9.32 D. However, corneal biomechanics are obviously changed after DALK surgery, which may explain the absence of the normal average flattening. We also found a good rotational stability of the toric IOL, since in no case the misalignment of the main axis of the IOL with respect to the 0–180° axis was higher than 10° at the last follow-up. These results are in good agreement with previous studies on toric IOLs only in eyes with PK [11, 12]. As a secondary outcome, we observed that all surgeries were safe and well tolerated by the corneal endothelium. During the follow-up period, there were no postoperative complications of the grafts. No episodes of immune-mediated rejection or other complications potentially compromising the VA were recorded [30].

Readers may be concerned about the mean age of our sample (67.1 ± 7.3 years), as this means that our patients did not undergo DALK in their 20s or 30s, as it usually happens, but in their 50s or 60s. The surgical indication at this age was mainly due to contact lens intolerance or progressive visual impairment.

This study has some limitations that warrant further investigations. First, the sample size was small. Second, we compared the preoperative TCA to the postoperative refractive astigmatism in order to evaluate the reduction of the astigmatism magnitude. This is not an ideal method because it compares values obtained from different measurements (Scheimpflug imaging of the cornea versus refraction). However, due to the presence of cataract, the measurement of the preoperative refractive astigmatism would not be reliable, so that total corneal astigmatism seems to be the best parameter for comparisons. In conclusion, our results suggest that toric IOL implantation after cataract surgery in patients previously treated with DALK represents a safe and effective procedure for the correction of astigmatism.

## Figures and Tables

**Table 1:** Pre- and postoperative operative clinical data of patients.

	Sim K	TCP	TCA power (D)	Axial length (mm)	DCVA (LogMAR)	UDVA (LogMAR)
Preoperative	45.37 (*±*2.19);	44.50 (*±*2.33);	4.92 (*±*1.99);	26.84 (*±*2.16);	0.55 (*±*0.29);	1.53 (*±*0.54);
	45.09;	41.52;	4.47;	27.09;	0.45;	1.65;
	42.71–49.29	43.99–47.89	2.66–9.32	22.6–29.7	1.0–0.2	2.0–0.7

Postoperative	45.54 (*±*2.19);	44.43 (*±*2.22);	5.08 (*±*2.76);	N/A	0.14 (*±*0.12);	0.29 (*±*0.13);
	^*∗*^43.16;	^*∗*^41.22;	^*∗*^4.21;		^*∗∗*^0.15;	†0.3;
	44.94–49.31	44.19–47.83	3.52–12.55		0.3–0	0.5–0

Sim K = simulated keratometry; TCP = total corneal power measured by ray-tracing; TCA = total corneal astigmatism; DCVA = distance-corrected visual acuity; UDVA = uncorrected distance visual acuity; IOL = intraocular lens. Each entity is reported as average (±SD); median; minimal value–maximal value. ^*∗*^Not statistically significant. ^*∗∗*^*p*=0.0020; †*p*=0.0039.

**Table 2:** Meridional and torsional power of preoperative and postoperative total corneal astigmatism and the surgically induced corneal astigmatism (SICA).

	Meridional power	Torsional power
Preoperative	−0.11 *±* 3.60; 0.52; −4.87 to 4.38	−1.93 *±* 3.72; −2.71; −6.70 to 3.48
Postoperative	0.16 *±* 4.10; −0.32; −5.72 to 7.38	−1.70 *±* 4.03: −2.38; −10.15 to 3.16
*p* value	0.6953	0.6953
SICA	0.27 *±* 1.16; 0.28; −0.86 to +3.00	0.23 *±* 1.44; 0.12; −2.00 to +2.42

SICA is defined as the difference between preoperative and postoperative total corneal astigmatism. Each entity is reported as average (±SD); median; minimal value–maximal value. All units are in diopters (D).

## Data Availability

All relevant data are available from the “Fondazione G.B. Bietti” for researchers who meet the criteria for access to confidential data.
